# Viral suppression rate at operation triple zero (Otz) and regular art follow-Up programs and associated factors among adolescent clients of Addis Ababa Ethiopia: a comparative cross-sectional study

**DOI:** 10.1186/s12985-023-02176-y

**Published:** 2023-09-08

**Authors:** Getahun Wedaje Tafere, Fufa Hunduma, Aman Yesuf

**Affiliations:** 1 Department of Pediatrics, St. Paul’s Hospital Millennium Medical College, Addis Ababa, Ethiopia; 2 School of public health, St. Paul’s Hospital Millennium Medical College, Addis Ababa, Ethiopia

**Keywords:** HIV in adolescents, Viral suppression, Operation triple zero (OTZ), Comparative cross-sectional study

## Abstract

**Background:**

- Viral suppression is the main goal of currently available treatment and it is used as a primary indicator of successful treatment for human immunodeficiency virus/acquired immune deficiency syndrome (HIV/AIDS). This ensures a reduction in AIDS-associated morbidity and mortality and decreases the risk of both vertical and horizontal viral transmission. This study aimed to investigate the magnitude of viral suppression and its associated factors among adolescents, comparing the operation triple zero (OTZ) program to regular antiretroviral therapy (ART) follow-up programs.

**Methods:**

- The research consisted of a comparative cross-sectional study, which included a total sample size of 446 adolescents on Active Antiretroviral Therapy (ART) — 223 enrolled in OTZ, and 223 in regular ART from public hospitals. Sociodemographic data were obtained using a self-administered semi-structured questionnaire, and clinical data were extracted from medical records. To examine the prevalence of viral suppression (VS) the latest status was used and multivariate logistic regression analysis was performed to identify factors associated with VS.

**Results:**

- Overall, the adolescent viral suppression was 88.3%, with 92.4% in the OTZ group and 84.3% in the regular ART group. Among OTZ participants, the factors that significantly reduced the odds of viral suppression were having a history of admission in the last year, alcohol use, biological mother not alive, cigarette smoking, social discrimination, and current medication regimen TDF/3TC/EFZ. Among those in regular ART, factors associated with a lesser chance of viral suppression were alcohol use, social discrimination, unscheduled appointments, and current medication regimen TDF/3TC/EFZ when compared to their counterparts. When we compared the two programs, OTZ had a 26.1 times greater chance of suppressing HIV viral load (AOR = 26.1, 95% CI: 3.499–133.9; P = 0.041).

**Conclusion:**

- The overall VS was 88.3%, while viral suppression is better achieved through the OTZ program than through regular ART services. Alcohol use, biological mother not alive, cigarette smoking, social discrimination, and current medication regimen TDF/3TC/EFZ were identified to be factors associated with viral nonsuppression. Therefore, it is advisable to implement the OTZ program in all HIV care centers and focus on factors reducing viral suppression.

## Introduction

Human immunodeficiency virus/acquired immune deficiency syndrome (HIV/AIDS) remains a major public health problem globally [1]. In 2022 it claimed the lives of 630 000 [480 000–880 000] and 1.3 million people were newly infected with HIV [2]. The majority of the burden of the infection and the disease occurs in Africa; Specifically in sub-Saharan countries [2, 3].

Adolescents and young adults account for 40% of new HIV infections globally and are a socially and economically vulnerable population. It is a period of exploration and experimentation. The adolescent age group is the most sexually active, especially between the age of 15 and 19 years old [4]. Many Young people are vulnerable to HIV at two stages of their lives; in the first decade of life when HIV can be transmitted from mother to child which is vertical transmission and the second decade of life when adolescence brings new vulnerability to HIV. In 2016, 55,000 adolescents between the ages of 10–19 died from AIDS-related causes. Daily, there are up to 150 adolescent deaths from AIDS-related illnesses globally, with 91% of these deaths occurring in sub-Saharan Africa [5].

Targeting adolescents and young adults for HIV prevention and treatment is essential for any national program’s success [4, 6–8]. In 2019, approximately 1.7 million adolescents aged between 10 and 19 years old were living with HIV globally, and around 190,000 new cases of HIV were reported among this age group [9]. In 2022, there were approximately 480,000 new cases of HIV among young people aged between 10 and 24 years old globally. Of these cases, around 140,000 were adolescents aged between 10 and 19 years old [10, 11].

In Ethiopia, it has been found that the prevalence of HIV among adults aged 15 to 59 is 0.9%. However, this prevalence varies depending on factors such as gender, age, and other demographic characteristics. It is worth noting that urban areas have an HIV prevalence (2.9%) compared to rural settings (0.4%). And women have twice the prevalence of HIV (1.2%) compared to men (0.6%). In Addis Ababa, the prevalence of HIV was found to be 3.4% [12–14].

The treatment for HIV involves the use of therapy (ART) to manage the virus [16–18]. It is recommended for everyone diagnosed with HIV to start ART as soon as possible. Although ART cannot cure HIV it significantly improves the lifespan and overall health of individuals living with the virus by suppressing the viral load in the blood. Typically, an initial HIV treatment plan consists of taking three HIV medicines from at least two different drug classes [18, 19]. The effectiveness of treatment can be monitored by measuring the viral load in the blood [15]. To track the effectiveness of treatments in patients receiving ART the usual method involves monitoring the HIV load over some time [16]. As per the guidelines provided by WHO, virological failure is detected when patients consistently have a load exceeding 1000 copies/ml after being on ART for 6 to 12 months [16, 17].

Maintaining undetected viral suppression or low viral load is a crucial goal of treatment for people living with HIV (PLHIV) for both clinical and transmission prevention benefits [4, 18]. According to the recommendations of the World Health Organization, individuals who start antiretroviral treatment (ART) should undergo viral load monitoring at six and twelve months after initiation. Afterward, stable individuals should have annual viral load monitoring [15].

The prevalence of viral suppression among HIV-positive adolescents in urban Ethiopia is lower than intended, at 48.2% [19, 20]. To achieve the goal of viral suppression, Ethiopia has initiated Operation Triple Zero (OTZ) programs in collaboration with ICAP and CDC [21].

The Operation Triple Zero (OTZ) program aims to achieve three key zeroes, namely, zero missed appointments, zero missed medications, and zero viral loads. The program promotes other zeroes, including zero stigma, zero admission, zero death, and zero unprotected sexual activity for sexually active adolescents [21, 22]. The program also promotes zero mother-to-child HIV transmission for pregnant and breastfeeding adolescents and young people living with HIV. To implement these strategies, the program engages adolescents, adolescents living with HIV, and adolescent peer groups in pediatric ART clinics in clubs, songs, dances, and peer support to empower them and improve their quality of health [22]. OTZ provides weekend services, monthly peer support group sessions, integrated sexual and reproductive health services, HIV status disclosure counseling, and high viral load management. This helps to manage the anxiety of adolescents and encourages them to follow up and drug adherence [21, 22].

Due to the regular ART care lags behind the goal of the country towards viral suppression, the OTZ program has been implemented in Ethiopia as a pilot program in five public hospitals, including St. Paul’s Hospital Millennium Medical College (SPHMMC), Zewditu Memorial Hospital(ZMH), Alart Hospitals, Yekatit 12 Hospital, and Black Lion Hospital. These hospitals are tertiary hospitals and have been providing ART services since the introduction of it to the country. Currently, they are providing both OZT and regular ART programs simultaneously. However, there is limited data and knowledge regarding the performance of the OTZ program when compared to the regular ART program. Therefore, this study aims to compare the efficacy of OTZ and regular ART programs regarding viral suppression and identify the prevalence and associated factors of viral suppression among adolescents on ART at OTZ centers and regular ART clinics.

## Methods and materials

### Study design

A comparative cross-sectional study design was used to assess the level of viral suppression and associated factors among HIV-infected adolescents among OTZ and regular ART programs. Addis Ababa, Ethiopia 2021.

### Study setting and period

The research was conducted at Saint Paul’s Hospital Millennium Medical College, Black Lion Hospital, and Zewditu Memorial Hospital, all of which have been implementing regular ART (RART) programs and practicing the Operation Triple Zero (OTZ) program since 2018. The OTZ program is also being provided at Yekatit 12 and Alert Hospitals. Out of the facilities providing OTZ program services, Black Lion Hospital, Zewditu Memorial Hospital, and Saint Paul’s Hospital Millennium Medical College (SPH) have the largest proportion of ART clients. Black Lion Referral Hospital is the largest teaching hospital in Ethiopia, while Zewditu Memorial Hospital is a specialized hospital that provides maternal and child health services and offers the largest number of ART services. Saint Paul’s Hospital Millennium Medical College, the second largest hospital in Ethiopia, is also a well-known HIV/AIDS treating, preventing, and controlling setting. All of these hospitals are located in Addis Ababa, the capital city of Ethiopia. Finally, the study period was conducted from July 1st to 30th, 2021.

#### The inclusion criteria were as follows

all HIV-positive adolescents who were on antiretroviral therapy (ART) and were being followed up in the Operation Triple Zero (OTZ) program. In addition, they should have attended the adolescent ART clinic for at least six months. Only adolescents between the ages of 10 and 19 years old will be included.

**The exclusion criteria** were adolescents who had been on ART for less than six months and had been enrolled in the OTZ clinic for less than six months. Additionally, those who are less than 10 years or over 19 years old will also be excluded from the study.

### Sample size determination

The determination of the sample size for the research study was computed using Epi Info Version 7.2.1.0 software, utilizing a comparative cross-sectional study design. To achieve this, several assumptions were considered, which included a 95% confidence level, 80% power, 5% margin of error, the ratio of exposed to unexposed (1:1) and percent of an outcome in unexposed to be 81% and percent of an outcome among exposed 91% (percent of the outcome in exposed and unexposed was taken from a previous report [21]. Being included in the OTZ program was considered an exposure. We got 416 from the assumptions and added a 5% non-response rate to a get final sample size of 446, with equal distribution between OTZ and regular ART centers.

### Sampling technique/procedures

From a total of five centers providing both OTZ and RART, a multistage (two-stage) sampling method was employed to include three study centers, namely, Black Lion Hospitals (BLH), Zewuditu Memorial Hospital (ZMH), and Saint Paul Hospital Millennium Medical College (SPHMMC). These centers were selected randomly to control any selection bias. Thereafter, proportional-to-size, allocation was computed to include participants from each selected hospital. All participants receiving services at the OTZ centers and regular ART centers were assigned equal proportions. Finally, each participant in each center was selected using a simple random sampling technique (lottery method).

**Fig. 1:**
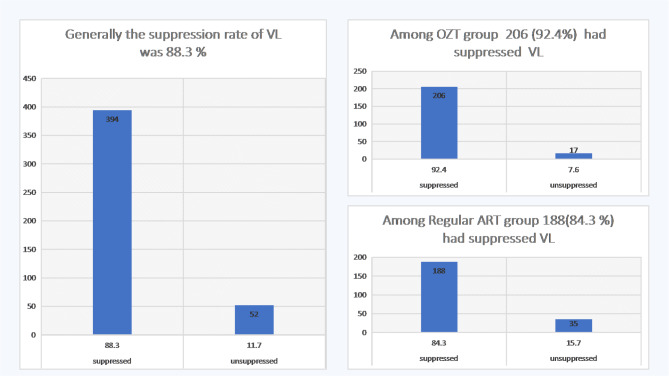
Rate of viral suppression overall, at regular ART(RART) program and OZT program, Ethiopia, 2021

Consequently, there were a total of 922 adolescents living with HIV clients in the three selected hospitals, with 325 (n1), 285 (n2), and 312 (n3) from Zewditu Memorial Hospital (ZMH), Saint Paul’s Hospital Millennium Medical College (SPHMCC), and Tikur Ambessa Hospital (TAH), respectively. The total required sample size for the project was 446 (n), with 223 allocated to the OTZ centers and the remaining 223 allocated to the regular ART centers. Participants from OTZ and regular ART were allocated equally (n) for each institution (Fig. 1).

Operation triple zero (OTZ) = 223.

Regular ART (RART) = 223.

Accordingly: $ZMH\left({n}_{1}\right)=\frac{n}{N}\times {N}_{1}$this is equal to$n=\frac{446}{922}\times 325=157$

Subsequently, participants for OTZ and regular ART are allocated equally n1 ÷ 2 = *n1*,

$\frac{157}{2}=79$ Seventy-nine OTZ clients and 79 regular ART clients will be selected.

The next is the same procedure:

$$\eqalign{\,SPHMMC(N2) & = \frac{n}{N} \times N2\\& = \frac{{446}}{{922}} \times 285 = 138\,then\,\frac{{138}}{2} = 69}$$

$$BLRH(n3) = \frac{n}{N} \times N3 = \frac{{446}}{{922}} \times 312 = 151\,then\,\frac{{151}}{2} = 76$$

### Dependent variables

Viral suppression.

### Independent variable

The independent variables used to assess the prevalence and associated factors of viral suppression for HIV-infected adolescents are classified as follows:

i. Demographic characteristics of age, sex, religion, ethnicity, income, schooling.
ii. Behavioral factors alcohol consumption, chat chewing, sexual behaviors, cigarette smoking.
iii. Clinical care/factors of appointment viral load status, adherence.
iv. Psychosocial factors- stigma, discrimination, peer support, parental alive/died.

### Operational definition of terms

Virological suppression: Virologic suppression is achieved when the HIV plasma viral load is less than 1,000 copies/ml based on one viral load measurement after six months on ART [23, 24]. Adherence: Adherence is assessed by pill count and child/caretaker’s self-report. Good adherence is considered when the patient misses less than or equal to 2 of 30 prescribed doses. Fair adherence is considered when the patient misses 3–5 of 30 prescribed doses. Poor adherence is considered when the patient misses less than 6 of 30 prescribed doses [25, 26]. Operation triple zero (OTZ) is a program of comprehensive HIV treatment to achieve “three zeroes”: zero missed appointments, zero missed drugs/medications, and zero viral loads (VLl) [21]. Discrimination in HIV is the act of treating people living with HIV differently than those without HIV. It can take various forms, such as refusal of health care, social isolation, verbal abuse, or rejection [27]. Moderate drinking is up to 1 drink per day for women and up to 2 drinks per day for men. One drink is a 12-ounce bottle of beer, a 5-ounce glass of wine, or a shot of liquor [28]. In this study, smoking was defined as smoking one stick of cigarette at least once per day.

### Viral load determination procedures

Approximately 10ml of whole blood was taken and divided into two EDTA tubes, with 2ml transferred to another tube and left at room temperature for 24 h. The remaining 8ml of blood was centrifuged at 5000 rpm for 5 min, resulting in 0.35ml of plasma which was then divided into six 1.8ml Thermo Scientific NunC tubes. These six plasma samples were stored under three different temperature conditions: two at room temperature, two at 2–8 °C, and two at -20 °C. One aliquot with a volume of 0.4ml was diluted in different concentration ratios (1:2, 1:3, and 1:5) as per standard. The results obtained from the diluted samples were adjusted using dilution correction factors before conducting data analysis. All diluted plasma samples were tested within six hours of collection. The remaining 1.5ml aliquot was subjected to a four-cycle freeze-thaw experiment. The real-time HIV-1 viral load assay was performed using the M2000 SP/RT platform by Abbott Molecular, following the manufacturer’s instructions [29].

### Data collection tools and procedures

Three data collectors were hired in the respective hospitals for data collection. For data collection tools and procedures, a pretest was conducted using a self-administered questionnaire assisted by family or guardians, and a standard checklist was used to review charts. The questionnaire drew on components from different literature sources, in addition to using the OTZ register book. It aimed to gather a range of demographic, behavioral, clinical, and psychosocial variables to help measure associated factors and the viral suppression rate among HIV-infected adolescents at ART and OTZ centers.

### Data quality

To maintain data quality, the questionnaires were initially translated into the local Amharic language and then retranslated back into English by different translators for consistency. Data collectors received training on confidentiality maintenance, data privacy, patient information collection, and patient chart review. The questionnaire was pretested on a sample of 5% of the total size in other hospitals. All completed questionnaires were checked for completeness, accuracy, and consistency, and any necessary corrections were made promptly. The principal investigator supervised all data collection activities.

### Data entry and analysis

For data entry and analysis, EPI-INFO version 7.2.1.0 and SPSS version 20 were used. To describe the demographics of ART clients, descriptive statistics methods such as frequencies, percentages, and mean/standard deviation were applied to the data from both programs separately. The prevalence rate of viral load (VL) suppression was calculated separately for each program, and a t-test was used to check the mean difference in viral suppression. In the initial model, bivariate analysis was conducted to identify variables that were associated with the outcome variable at p = 0.20, and these variables were included in the final model. The final model used multivariate binary and multiple logistic regression to identify factors associated with viral suppression. Adjusted odds ratios with a p-value of less than or equal to 0.05 and 95% confidence intervals were considered significant in declaring association.

### Ethical considerations and consent to participate

A letter was primarily received from SPHMMC, Department of Public Health. In addition, the proposal was submitted for review, and further ethical approval was given by SPHMMC- Institutional Review Board with letter number PM23/278. Next, ethical approval from the IRB of each study setting of this project was received. Written assent was obtained from each study participant and/or family and/or guardian after explaining the objective of the study. Participation of the respondents was volunteer-based based and kept any privacy issues confidential. Cultures and norms were respected properly. Each participant can be withdrawn at any time during the interview process. No names were used when the questionnaires were serial numbers only for the purpose of confidentiality. Finally, we approve that this research was done according to the Declaration of Helsinki Ethical Principles.

## Results

### Sociodemographic characteristics of participant adolescents on HAART at public health hospitals of Addis Ababa, Ethiopia 2021

The study included a total of 446 individuals, with 223 enrolled in the OTZ program and the remaining 223 in regular ART. Of the participants, 54.3% were female and 45.1% were male. All respondents were aged between 10 and 19 years old. When examining the programs separately, it was found that 55.3% of OTZ participants were female, with an age distribution of 23.3% aged 10–13 years old, 52.5% aged 14–16 years old, and 24.2% aged 17–19 years old. The majority of participants in both programs had completed secondary school, with 67.3% of OTZ participants and 46.6% of regular ART participants at this level of education. The most common religion among respondents was Orthodox, with 69.5% of adolescents identifying as such. Furthermore, a significant proportion of participants reported that their biological mother (55.3%) or father (57.5%) had passed away (Table 1).

**Table 1:** Sociodemographic characteristics of study participants among adolescents on ART services at public health hospitals Addis Ababa 2021

Variables	CCategory	OTZ (n = 223)		RART (n = 223)	
		frequency	percent	frequency	percent
Sex	Male Female	102	45.7	98	43.9
		121	54.3	125	56.1
Age	10–13	52	23.3	112	50.2
	14–16	117	52.5	38	17.0
	17–19	54	24.2	73	32.7
Educational status	primary school	150	56.1	132	59.2
	secondary school	57	37.7	56	25.1
	preparatory school	16	6.3	35	15.7
	no education	-	-	-	-
Religion	orthodox	155	68.2	151	67.7
	Muslim	37	17.0	49	22.0
	catholic	10	5.4	4	1.8
	protestant	16	7.2	16	7.2
	others	6	2.2	3	1.3
Your Biological parents’ alive? mother	yes	100	44.8	73	32.7
	No	123	55.2	150	67.3
Father	yes	94	42.2	69	30.9
	no	129	57.8	154	69.1
Living biological parents	With Only my mother	33	70.0	26	11.7
	With only father	13	29.1	17	7.6
	with both mother &father	66	0.9	37	16.6
	not at all	111	70.0	143	64.1
Having history of admission in the last year	yes	33	14.8	8	3.6
	No	190	85.2	215	96.4

OTZ = Operation Triple Zero, RART = regular antiretroviral therapy

Among Otz participants, 15.7% reported using alcohol, 4.5% smoked cigarettes, and 0.9% used chewing chat. Additionally, 6.3% experienced social stigma, and 4.9% experienced social discrimination. However, the vast majority (91.9%) reported having peer support related to their health problem. In contrast, among regular ART participants, 14.8% reported using alcohol, 1.8% smoked cigarettes, and 0.9% used chewing chat. Similarly, 6.3% experienced social stigma, 4.9% experienced social discrimination, and 93.7% reported having peer support related to their health problem (Table 2).

**Table 2:** Sociobehavioral factors of study participants among adolescent ART clients at public health hospitals, Addis Ababa, 2021

Variables	Categories	OTZ (n = 223)		RART (n = 223)	
		frequency	percent	frequency	percent
Are you drink alcohol	yes	35	15.7	33	14.8
	No	188	84.3	190	85.2
Smoke cigarette	yes	10	4.5	4	1.8
	No	213	95.5	219	98.2
Chewing chat	yes	2	0.9	2	0.9
	No	221	99.1	221	99.1
Did you have got a stigma	yes	12	5.4	14	6.3
	No	211	94.6	211	93.7
Discrimination	yes	10	4.5	11	4.9
	No	213	95.5	212	95.1
Have you peer support related to your health problem?	yes	205	91.9	209	93.7
	No	18	8.1	14	6.3

OTZ = Operation Triple Zero, RART = regular antiretroviral therapy

### Medical and adherence characteristics of study participants

Among the participants of the OTZ program, the most prevalent duration of ART was between 11 and 15 years, comprising 129 individuals (57.8%). The majority of OTZ respondents, 160 individuals (71.7%), were currently taking the TDF, 3TC, and DTG medication regimen. Of these individuals, the majority, 162 (72.6%), reported good adherence, while 13 (13.5%) reported fair adherence, and 47 (47.5%) reported poor adherence to the medication. Additionally, more than half of the OTZ participants (58%) followed their appointment schedule, while the remaining 93 (41.9%) did not. Furthermore, 70% of OTZ participants had taken INH-TB prophylaxis within the prior year (Table 3).

**Table 3:** Clinical characteristics of study participants among adolescent clients on ART at public health hospitals, Addis Ababa 2021

Variables	Categories	OTZ (n = 223)		RART (n = 223)	
		frequency	percent	frequency	percent
Duration of on ART in year	7–11 month	-	-	-	-
	1–5	4	1.8	10	4.5
	> 5–10	61	27.4	124	55.6
	> 11–15	129	57.8	36	16.1
	> 15	29	13.0	53	23.8
The last appointment	schedule	130	58.3	94	42.2
	unscheduled	93	41.7	129	57.8
level of viral load	undetectable	178	79.8	152	68.2
	low detectable	12	5.4	12	5.4
	151 Abbott or 21 ccppt-1000copy/ml	16	7.2	24	10.8
	high viral load ≥ 1000 copy	17	7.6	35	15.7
Current medication	ABC/3TC/ LOP/r	18	8.1	34	15.2
	AZT/3TC/ LOP/r	4	1.8	22	9.9
	TDF/3TC/DTG	160	71.7	98	43.9
	TDF/3TC/ EFZ	8	3.6	22	9.9
	Other combination	33	14.8	47	21.1
Adherence	Good	162	72.6	162	72.6
	Fair	14	6.3	13	5.8
	Poor	47	21.1	48	21.5
TB prophylaxis in the last years	Yes	156	70	101	45.3
	No	67	30	120	53.8
mean viral load		4477.2		6269.7	

RART = regulr antiretroviral therapy, ABC/3TC/ LOP/r = abacavir/lamivudine/lopinavir/ritonavir, AZT/3TC/ LOP/r = abacavir/lamivudine/lopinavir/ritonavir, TDF/3TC/DTG = tenofovir disoproxil fumarate/lamivudine/dolutegravir, TDF/3TC/ EFZ = tenofovir disoproxil fumarate/lamivudine/efavirenz

### Rates of viral suppression

Overall, the suppression rate was found to be 88.3%, (n = 394). The suppression rate was higher among adolescents who were included under the OTZ program; 92.4% (n = 206) and 84.3% (n = 188) among those who were under the regular ART program (Fig. 1).

In comparison, among regular ART participants, the prevalent duration of HAART was between 5 and 10 years, with 124 individuals (55.6%). The most common regimens for HAART were TDF, 3TC, and DTG, which were taken by 98 individuals (43%). Similar to the OTZ program participants, the majority of regular ART participants, 162 individuals (72.6%), reported good adherence, while 13 (5.8%) had fair adherence, and 48 (21.5%) had poor adherence. Furthermore, 129 individuals (57.8%) out of the regular ART participants came to the ART clinic unscheduled at their last appointment, while approximately half of the participants (53%) had taken INH-TB prophylaxis within the past year (Table 3).

### Factors associated with viral suppression in the operation triple zero program

The initial model (bivariate logistic regression) included several variables, such as educational status, father’s survival, living arrangements of parents, history of admission, alcohol consumption, cigarette smoking, biological mother’s survival, social discrimination, social stigma, and current medication regimen. These variables were included in the final model after identification based on preset criteria. Multivariate analysis was conducted, and it was found that having a history of admission in the last year decreased the odds of viral suppression by 53% (adjusted odds ratio (AOR) = 0.47; 95% CI: 0.015–0.157; P = 0.001), and alcohol use decreased the odds of viral suppression by 98.5% (AOR = 0.015; 95% CI: 0.001–0.206, P = 0.002). Moreover, those who had their biological mother deceased had a decreased odds of viral suppression by 91.7% (AOR = 0.083; 95% CI: 0.010–0.730; P = 0.025), while cigarette smokers had a decreased chance of viral suppression by 98% (AOR = 0.02; 95% CI: 0.001-0.370; P = 0.009). Additionally, social discrimination decreased the chance of viral suppression by 95.6% (AOR = 044, 95% CI:0.003–0.692; P = 0.026), and the current medication regimen of TDF, 3TC and EFV decreased the chance of viral suppression by 95.6% (AOR = 0.044; 95% CI:0.003–0.692; P = 0.002) when compared to their counterparts, as observed in Table 4.

**Table 4:** Bivariate and multivariable analyses of factors associated with viral suppression among adolescent OTZ patients (n = 223) at a public hospital, Addis Ababa, 2021

Variable	category	viral suppression status (n = 223)		COR (95% CI)	AOR (95% CI)	P value
		Suppressed	Unsuppressed			
Having history of admission in the last 1 year	Yes	21	12	0.047(0.015 − 0.147)	0.01(0.001-0.157)	0.001*
	No	185	5	1	1	
Alcohol use	yes	27	8	0.17(0.06–0.478)	0.015(0.001-0.206)	0.002*
	No	179	9	1	1	
smoke cigarette	Yes	2	8	0.011(0.002 − 0.06)	0.02(0.001-0.370)	0.009*
	No	204	9	1	1	
Biological mother alive	yes	97	3	0.241(0.067-0.863)	0.083(0.01-0.731)	0.025*
	No	109	14	1	1	
Discrimination	yes	5	6	0.046(0.012-0.173)	0.044(0.003-0.692)	0.026*
	No	201	11	1	1	
Current medication Regimen	ABC/3TC LOP/r	18	0	--	---	.--
	AZT/3TC LOP/r	4	0		-	--
	TDF/3TC/DTG	153	7	1	1	
	TDF/3TC/EFZ	7	1	0.143(0.048- 0.429)	0.025(0.003-0.246	0.002*
	Other combination	31	2	0.742(0.174 -6.223)	0.144(0.001-41.57)	0.503

*p value < 0.05 significant associated., VL = viral load, ABC/3TC/ LOP/r = abacavir/lamivudine/lopinavir/ritonavir, AZT/3TC/ LOP/r = abacavir/lamivudine/lopinavir/ritonavir, TDF/3TC/DTG = tenofovir disoproxil fumarate/lamivudine/dolutegravir, TDF/3TC/ EFZ = tenofovir disoproxil fumarate/lamivudine/efavirenz, AOR = adjusted odd ratio

### Factors associated with viral suppression among adolescents in the regular ART (RART) program

The initial model revealed that age (10–13 years), level of education (primary school), history of admission in the last year, alcohol use, cigarette smoking, having a living biological mother, experiencing social stigma, and current medication regimen (TDF, 3TC, DTG, and other combinations) were associated with viral load (VL) suppression among those enrolled in the regular ART program. However, after adjusting for possible confounding, certain factors were found to significantly affect viral suppression. Specifically, alcohol use decreased viral suppression by 96% (AOR = 0.038, 95% CI: 0.006–0.240; P = 0.001); social stigma decreased viral suppression by 99% (AOR = 0.010, 95% CI: 0.001–0.142; P = 0.043); having a living biological mother increased viral suppression by nine times (AOR = 8.9, 95% CI: 1.075–73.627; P = 0.005); unscheduled appointments decreased the chance of viral suppression by 95% (AOR = 0.043, 95% CI: 0.005–0.396; P = 0.001); the current medication regimens TDF/3TC/EFZ decreased viral suppression by 94% (OR = 0.06, 95% CI: 0.009–0.389; P = 0.003) compared to their counterparts. (Table 5).

**Table 5:** Bivariate and multivariable analyses of factors associated with viral suppression among adolescents undergoing regular ART (n = 223) at a public hospital, Addis Ababa, in 2021

Variable	Category	viral suppression status (n = 223)		COR (95% CI)	AOR (95% CI)	P value
		Suppressed	Unsuppressed			
Alcohol use	yes	20	13	0.201(0.088–461)	0.038(0.006-0.240)	0.001*
	No	168	22	1	1	
Discrimination	yes	3	11	0.035(0.009-0.136)	0.010(0.001-0.142)	0.043*
	No	185	24	1	1	
Biological mother alive	yes	71	2	10(2.331–43.0)	8.9(1.075–73.627)	0.005*
	No	117	33	1	1	
the last appointment	schedule	93	1	1	1	
	unscheduled	95	34	0.33(0.004–0.022)	0.043(0.005-0.396)	0.001
current medication regimen	ABC,3TC, LOP/r	33	1	3.751 (0.981–5.241)	2.392 (0.854–4.259)	0.081
	AZT,3TC, LOP/r	22	0	--	--	--
	TDF/3TC/ DTG	88	10	1	1	.
	TDF/3TC/EFZ	10	12	0.091(0.131-0.837)	0.06(0.009-0.389)	0.003*
	Other combination	35	12	0.331(0.0207-10.153)	0.151(0.045-6.821)	0.574

ABC/3TC/ LOP/r = abacavir/lamivudine/lopinavir/ritonavir, AZT/3TC/ LOP/r = abacavir/lamivudine/lopinavir/ritonavir, TDF/3TC/DTG = tenofovir disoproxil fumarate/lamivudine/dolutegravir, TDF/3TC/ EFZ = tenofovir disoproxil fumarate/lamivudine/efavirenz

### Factors associated with viral suppression among adolescents in both the regular ART and OTZ programs

After merging patients from both programs, bivariate analysis showed that OTZ participants, history of admission in the last year, alcohol and cigarette use, having a living biological mother, social discrimination, unscheduled appointments, and current medication regimen was associated with viral suppression. However, after adjusting using a multivariable regression statistic, participants enrolled in the OTZ program had a 21.6-fold chance of having suppressed viral load (adjusted odds ratio [AOR] = 21.6, 95% CI: 3.499–133.9; P = 0.041). Those who experienced social discrimination were 97.6% less likely to experience VS (AOR = 0.024, 95% CI: 0.003–0.183; P = 0.001), while those who had a living biological mother had a 7.5 times higher chance of viral suppression (AOR = 7.5, 95% CI: 1.502–37.491; P = 0.017). Those who smoke cigarettes had 99.2% less likelihood of viral suppression, (AOR = 0.008, 95% CI: 0.000–0.359; P = 0.002 ) and those who drink alcohol had 98.8% less odd of viral suppression, (AOR = 012; 95% CI: 0.002–0.078). Sticking to scheduled appointments had an 18 times higher likelihood of viral suppression than those who came on unscheduled (AOR = 18, 95% CI: 4.120–34.271; P = 0.002). Tenofovir disoproxil fumarate/lamivudine/dolutegravir (TDF/3TC/EFZ) regimen had 97.5% less chance of viral suppression when compared to TDF/3TC/DTG regimen. (Table 6).

**Table 6:** Bivariate and multivariable analyses of factors associated with viral suppression among adolescent patients (n = 446) at the public hospital Addis Ababa, 2021

Variable	Categories	Viral suppression status (n = 446)		COR (95% CI)	AOR (95% CI)	P value
		Suppressed	Unsuppressed			
OTZ	Yes	206	17	2.26(1.223–4.16)	21.6(3.499–133.9)	0.041
	No	188	35	1	1	
Have hx of admission	Yes	22	19	0.103(0.051-0.2090	0.005(0.000- 0.050)	0.000
	No	372	33	1	1	
Alcohol use	Yes	47	21	0.20(0.106-0.376)	0.012(0.002- 0.078)	0.022
	No	347	31	1	1	
Smoke cigarette	Yes	4	10	0.043(0.013 − 0.143)	0.008(0.000 − 0.359)	0.008
	No	390	42	1	1	
Biological mother alive	Yes	168	5	6.7(2.72- 17.948)	7.5(1.502–37.491)	0.017
	No	226	47	1	1	
Discrimination	Yes	6	17	0.032(0.012- 0.086)	0.024(0.003 − 0.183)	0.001
	No	388	35	1	1	
last appointment	Schedule	22	2	32.3(7.743- 134)	18(4.120–34.271)	0.002
	Unscheduled	172	50	1	1	
Current medication regimen	ABC,3TC, LOP/r	51	1	3.59(0.820-4.541)	2.101(0.914.-11.271)	0.062
	AZT/3TC, LOP/r	26	0	--	--	---
	TDF/3TC/DTG	241	17	1	1	
	TDF/3TC/EFZ	16	14	0.082(0.0104-0.429)	0.025 (0.004 − 0.148)	0.000
	Other combination	60	20	0.263(0.091 − 6.3)	0.210(0.0850-14.484)	0.084

VL = viral load, ABC/3TC/ LOP/r = abacavir/lamivudine/lopinavir/ritonavir, AZT/3TC/ LOP/r = abacavir/lamivudine/lopinavir/ritonavir, TDF/3TC/DTG = tenofovir disoproxil fumarate/lamivudine/dolutegravir, TDF/3TC/ EFZ = tenofovir disoproxil fumarate/lamivudine/efavirenz

## Discussion

This study has evaluated the overall viral suppression rate and compared the viral suppression rate among operation triple zero (OTZ) program and regular ART participants and identify associated factors with viral suppression among adolescents at selected public hospitals. The study revealed that there was 88.3% overall viral suppression at the hospitals. This is in line with other sub-Saharan high HIV infection load countries. According to CDC’s report, it is indicated that seven of eight sub-Saharan African countries (Côte d’Ivoire, Kenya, Lesotho, Malawi, Namibia, South Africa, Tanzania, and Uganda) achieved > 85% of viral suppression in 2018 [30]. However, this indicates that Ethiopia had lagged behind the target of adopting the UNAIDS “90-90-90” strategy to increase VS to at least 90% by 2020 [31]. Many African countries had also failed to achieve 2020’s 90-90-90 target except South Africa which achieved the goal in 2019 [32]. Therefore, these countries need to work extensively to catch up to 2025’s 95–95 -95 target, which OTZ could be one strategy.

This study indicated that the suppression rate of viral load was higher among patients on the OTZ program (206, 92.4%) than among adolescents on the regular ART program (188, 84.3%). A study performed in Kenya among adolescents aged 10–19 years who were enrolled in OTZ showed that viral suppression increased from 65 to 80% among those aged 10–14 years and increased from 66 to 84% among 15–19-year-olds. A comparative study conducted in South Africa indicated that viral suppression among adolescents and young adults attending the adolescent clinic was 91%, while that among adolescents attending the standard pediatric clinic was 80% [4]. This is also in line with what CDC estimates the viral suppression to be among Ethiopian OTZ patients which is more than 90% [20]. Operation triple zero improves viral suppression through zero missed appointments, zero missed antiretroviral drugs, and zero viral loads through peer-to-peer support programs, training, and OTZ championship [33, 34]. It provides youth-friendly HIV services, connectedness, positive living messages, caregiver education on supportive parenting, and strategies to improve health. This helps the adolescents to accept living with HIV peacefully [35].

Overall, those who were included in the OTZ program, their biological mother alive, and those who stick to scheduled follow-up had more chance of viral suppression when compared to their counterparts. OTZ program so far implemented and improved the viral suppression rate in countries like Kenya, Nigeria, and Zimbabwe [34, 35]. Therefore, OTZ could enable countries to reach 2025’s 95-95-95 target of viral suppression in those who need to improve their performance. A study done in Ghana indicates that the type of the guardian, or their gender, influences the perceived stigma and available social support in adolescents living with HIV [36]. It was also revealed in other studies that patients who have higher attendance of scheduled follow up has a higher chance of viral suppression [37]. This can be indirectly related to adherence, those who lost on their regular follow-up would run out of their pills.

Taking current medication TDF/3TC/EFZ, alcohol use, smoking cigarette, and facing social discrimination were associated with less likely VS than their counterparts. The study comparing DTG-based regimens with TDF/3TC/EFZ showed that the previous one has a superior VS rate [38]. Similar to this study, alcohol use, cigarette smoking, and discrimination were identified to be factors that are associated with a lesser chance of viral suppression [39–41]. In Ethiopia, about 37.63% of youth/high school students use at least one substance [42]. Unless necessary attention is given to adolescents who are living with HIV, they are likely to be affected by their peers’ behavior.

The study was about the evaluation of a new program consolidated in Ethiopia and some African countries to achieve 95% viral suppression by 2025. It can be projected to the countries which are adopting OTZ to increase their capabilities of VS. However, one of the limitations of this study was the classification of viral suppression which was established as < 1000 copies/ml, which is not applicable in some parts of the world. The study used a cross-sectional design, which may lead to a failure to determine causality due to temporal issues. A self-completed questionnaire was the second technique of data collection that can introduce recall bias.

## Conclusion and recommendation

The study indicates that the overall viral suppression was 88.2% which is lagging behind the 90% UNAIDS target by 2020. However viral suppression was better achieved through the OTZ program (92.4%) than through the regular ART program (84.3). Alive biological mother, sticking to a regular schedule improves viral suppression while taking current medication TDF/3TC/EFZ, alcohol use, smoking cigarette, and facing social discrimination were found to be risk factors for viral non-suppression. Therefore, to achieve better viral suppression among adolescents, Ethiopia and other countries which are not on the right track of viral suppression by 2025, better to adopt the OTZ program. In addition to this, factors that are found to be associated with lesser viral suppression should be given due attention by HIV/AIDS care facilities.

### Generalizability

This study is generalizable for all setups in the country and outside, which are employing and want to employ the newer program of OZT in HIV care.

## Declarations

We hereby declare that all source materials used for this research have been duly acknowledged.

## Data sharing statement

Any data and materials related to this study are available upon request from the Principal Investigator (email: fhunduma@gmail.com).

## Abbreviations

ALHIV: Adolescent living human immunodeficiency virus
VS: Viral suppression
OTZ: Operation triple zero
AYPLHIV: Youth living with HIV
IRB: Institutional Review Board
OTZ: Operation Triple Zero
SPHMMC: St. Paul’s Hospital Millennium Medical College
VL: Viral load
